# Association of triglyceride-glucose index and neutrophil-to-lymphocyte ratio with coronary artery disease

**DOI:** 10.1186/s12872-023-03564-6

**Published:** 2023-11-01

**Authors:** Bing Zhang, Aihong Peng, Shu Li, Fei Li, Jing Wan, Jinping Lu

**Affiliations:** 1 Department of Cardiology, Zhongnan Hospital of Wuhan University, No 169 Donghu Road, Wuchang District, Wuhan, 430071 Hubei Province China; 2 Department of Critical Care Medicine, Zhongnan Hospital of Wuhan University, No 169 Donghu Road, Wuchang District, Wuhan, 430071 Hubei Province China; 3 Department of General Practice, Zhongnan Hospital of Wuhan University, No 169 Donghu Road, Wuchang District, Wuhan, 430071 Hubei Province China

**Keywords:** Coronary artery disease, Triglyceride-glucose index, Neutrophil-to-lymphocyte ratio

## Abstract

**Objective:**

The present study aimed to investigate the association of triglyceride-glucose (TyG) index and neutrophil-to-lymphocyte ratio (NLR) with coronary artery disease (CAD), and evaluate the cumulative value of TyG index and NLR in identifying CAD, as well as the severity of CAD.

**Methods:**

This retrospective study enrolled 2867 patients who underwent coronary angiography (CAG) for the first time between January 2013 and June 2022 in Zhongnan Hospital of Wuhan University. There were 2109 patients with CAD and 758 patients without CAD. The CAD patients were divided into two groups based on the median of Gensini score (mild stenosis CAD group: Gensini score < 26 points; severe stenosis CAD group: Gensini score ≥ 26 points). To further evaluate the cumulative value of TyG index and NLR in identifying CAD and CAD severity, all patients were classified into four groups based on median of TyG index and NLR: (1) the control group: patients with low-TyG and low-NLR; (2) isolated high-NLR group: patients with low-TyG and high- NLR; (3) isolated high- TyG group: patients with high-TyG and low- NLR; (4) high-TyG combined with high-NLR group: patients with high-TyG and high- NLR.

**Results:**

Multivariate logistic regression analysis showed that both the TyG index and NLR were independent risk factors for CAD, and they were also independent risk factors for severe stenosis in CAD (P < 0.05). Compared with the low-TyG and low- NLR group, patients in high-TyG and high- NLR group had a 1.418 times higher odds ratio (OR) of having CAD and a 1.692 times higher OR of having severe stenosis in CAD in the multivariable logistic regression model. It is worth noting that the OR values of the high-TyG and high- NLR group were higher than those of the isolated high-NLR group and the isolated high- TyG group. The ROC analysis showed that the combination of the TyG index and NLR was superior to TyG index or NLR in predicting CAD and CAD severity.

**Conclusion:**

Compared to TyG index or NLR, the combination of the TyG index and NLR is beneficial to improve the diagnostic accuracy of CAD and CAD severity.

## Introduction

Coronary artery disease (CAD) is a chronic cardiovascular disease caused by coronary atherosclerosis. Due to population aging, urbanization, and unhealthy lifestyle, the incidence and mortality of CAD are increasing, which has become a serious public health problem [1, 2]. Inflammation and insulin resistance (IR) play important roles in the occurrence and development of atherosclerosis [3–6]. Inflammation is also closely related to IR [7]. On the one hand, chronic inflammation is a key pathological mechanism of IR [8]. Chronic inflammation can impair normal lipid accumulation, adipose tissue function, mitochondrial function, and cause endoplasmic reticulum stress, which lead to IR. On the other hand, some studies have shown that IR can exacerbate chronic inflammation [9]. While it is unclear which comes first, IR or inflammation, it is clear that there is a vicious cycle between inflammation and IR.

Recent studies have confirmed that the triglyceride-glucose (TyG) index is a reliable index for evaluating IR [10, 11]. Neutrophil-to-lymphocyte ratio (NLR) as a marker of inflammation, can reflect the degree of systemic inflammation. Both TyG index and NLR are associated with atherosclerosis. However, the cumulative value of TyG index and NLR in predicting CAD and CAD severity is still uncertain. This study aimed to further evaluate the value of the combination of the TyG index and NLR.

## Patients and methods

### Study population

This study population comprised 2867 patients who underwent coronary angiography (CAG) for the first time and hospitalized in the Department of Cardiology, Zhongnan Hospital of Wuhan University from January 2013 to June 2022. Exclusion criteria: (1) congenital heart disease, old myocardial infarction, heart failure, valvular heart disease and other cardiac history; (2) previous coronary intervention or coronary artery bypass grafting; (3) infectious diseases, hematological diseases, malignant tumors, thyroid diseases, severe hepatic or renal dysfunction; (4) autoimmune diseases or being treated with hormones or immunosuppressants; (5) surgical operation or severe trauma within 3 months. This study conformed with the declaration of Helsinki and was approved by the Ethics Committee of Zhongnan Hospital.

### Data collection and laboratory determination

Personal information such as age, sex, smoking history, and medical history, such as a history of surgery, hypertension, diabetes, infectious diseases and other diseases were obtained from the medical records. All patients were required to fast overnight and then venous blood samples were collected the next day early morning. Subsequently, laboratory parameters, such as neutrophil (NE) counts, lymphocyte (LY) counts, fasting plasma glucose (FPG), total cholesterol (TC), triglycerides (TG), high-density lipoprotein cholesterol (HDL-C), and low-density lipoprotein cholesterol (LDL-C) levels were measured in the Department of Clinical Laboratory in our hospital. TyG index and NLR were calculated as follows: TyG = ln[fasting triglycerides (mg/dL) × fasting plasma glucose (mg/dL)/2]; NLR = [neutrophil counts (×10^9^/L)/lymphocyte counts (×10^9^/L)].

### Coronary angiography and groups

CAG was performed by at least two experienced cardiovascular interventional experts based on the Judkin method through the radial or femoral artery. The severity of CAD was assessed using the Gensini scoring system. Based on the result of CAG, all patients were divided into CAD group and non-CAD group. Then, we divided the patients with CAD into two groups according to the median of Gensini score as follows: mild stenosis CAD group: Gensini score < 26 points; severe stenosis CAD group: Gensini score ≥ 26 points.

### Dichotomy of TyG index and NLR

We defined high-TyG level as TyG index ≥ 8.78, high-NLR level as NLR ≥ 2.46, based on the median of all patients in this study. All patients were classified into four groups according to their level of TyG index and NLR: (1) the control group: without high-TyG and without high-NLR level; (2) isolated high-NLR group: with high-NLR but without high-TyG level; (3) isolated high-TyG group: with high-TyG but without high-NLR level; and (4) high-TyG combined with high-NLR group: with both high-TyG and high-NLR levels.

### Statistical analysis

Data analysis was performed by SPSS 26.0 software (IBM Corp, Armonk, New York, USA). Categorical variables were described as counts and percentages. Continuous variables with normal distribution were described as the mean ± standard deviation, whereas variables with non-normal distribution were described as median and interquartile range (25-75%). The Chi-square test was used for categorical variables and the Student’s t-test, Mann–Whitney test or Kruskal–Wallis test was used for continuous variables. Bonferroni correction was used for multiple comparisons. Logistic regression analysis was applied to identify the risk factors for CAD and severe stenosis in CAD. The receiver operating characteristic (ROC) curve analysis was performed to evaluate the cumulative value of the combination of the TyG index and NLR in predicting CAD and CAD severity. A two- tailed p-value of < 0.05 was considered significant.

## Results

### Baseline characteristics

Table 1 shows the baseline characteristics of patients with CAD and patients without CAD. Compared with the non-CAD group, the patients in CAD group tended to be older and male and had higher NE counts, TG, FPG, TyG index, NLR values but lower LY counts and HDL-C values (all P < 0.05). Moreover, patients in the CAD group tended to have a history of smoking, hypertension and diabetes (all P < 0.05).

Table 2 shows the baseline characteristics of patients stratified by TyG index and NLR level. Compared with the control group, the percentages of CAD and severe stenosis in CAD in the other three groups were higher (P < 0.05), especially in the high-TyG combined with high-NLR group.

**Table 1:** Baseline characteristics of the study patients

Characteristics	non-CAD group (n = 758)	CAD group (n = 2109)	t/χ2 Value	P Value
Male (n,%)	397 (52.4)	1467 (69.6)	72.392	< 0.001
Age (years)	59.50 ± 9.91	62.66 ± 10.05	-7.452	< 0.001
Smoking (n, %)	185 (24.4)	789 (37.4)	42.041	< 0.001
Hypertension (n, %)	390 (51.5)	1363 (64.6)	40.749	< 0.001
Diabetes (n, %)	103 (13.6)	599 (28.4)	66.177	< 0.001
NE counts (×10^9^/L)	3.72 ± 1.46	4.72 ± 3.09	-8.586	< 0.001
LY counts (×10^9^/L)	1.71 ± 0.60	1.61 ± 0.59	4.057	< 0.001
TC (mmol/L)	4.45 ± 0.98	4.36 ± 1.11	1.953	0.051
TG (mmol/L)	1.67 ± 1.18	1.82 ± 1.51	-3.842	< 0.001
HDL-C (mmol/L)	1.18 ± 0.31	1.07 ± 0.27	9.834	< 0.001
LDL-C (mmol/L)	2.69 ± 0.85	2.69 ± 0.92	0.165	0.869
FPG (mmol/L)	5.82 ± 1.98	6.22 ± 2.21	-4.357	< 0.001
TyG index	8.76 ± 0.64	8.87 ± 0.64	-4.057	< 0.001
NLR	2.43 ± 1.51	3.46 ± 3.14	-8.687	< 0.001

Abbreviations: CAD, coronary artery disease; NE, neutrophil; LY, lymphocyte; TC, total cholesterol; TG, triglycerides; HDL-C, high-density lipoprotein cholesterol; LDL-C, low-density lipoprotein cholesterol; FPG, fasting plasma glucose; TyG, triglyceride-glucose; NLR, neutrophil-to-lymphocyte ratio

**Table 2:** Baseline characteristics of patients stratified by TyG and NLR level

Characteristics	low-TyG index		high-TyG index		Overall P Value
	low-NLR (n = 690)	high-NLR (n = 744)	low-NLR (n = 737)	high-NLR (n = 696)	
Male (n,%)	404 (58.6)	518 (69.6)^a^	452 (61.3)	490 (70.4)^a^	< 0.001
Age (years)	59.81 ± 9.59	64.07 ± 10.04^a^	61.20 ± 9.70	62.08 ± 10.10^a^	< 0.001
Smoking (n, %)	214 (31.0)	251 (33.7)	238 (32.3)	271 (38.9)^a^	0.010
Hypertension (n, %)	377 (54.6)	443 (59.5)	465 (63.1)^a^	468 (67.2)^a^	< 0.001
Diabetes (n, %)	115 (16.7)	121 (16.3)	233 (31.6)^a^	233 (33.5)^a^	< 0.001
TC (mmol/L)	4.17 ± 0.97	4.07 ± 0.99	4.70 ± 1.11^a^	4.60 ± 1.10^a^	< 0.001
TG (mmol/L)	1.00 ± 0.52	0.99 ± 0.50	2.81 ± 1.82^a^	2.50 ± 1.92^a^	< 0.001
HDL-C (mmol/L)	1.19 ± 0.29	1.16 ± 0.29	1.03 ± 0.27^a^	1.02 ± 0.23^a^	< 0.001
LDL-C (mmol/L)	2.57 ± 0.89	2.53 ± 0.87	2.83 ± 0.93^a^	2.83 ± 0.90^a^	< 0.001
FPG (mmol/L)	5.19 ± 0.82	5.47 ± 1.05	6.60 ± 2.42^a^	7.20 ± 2.91^a^	< 0.001
Gensini score	19.0 (10.0, 27.0)	22.6 (16.0, 36.8)^a^	24.0 (13.0, 32.8)^a^	28.0 (19.6, 42.0)^a^	< 0.001
CAD (n,%)	428 (62.0)	581 (78.1)^a^	522 (70.8)^a^	578 (83.0)^a^	< 0.001
CAD severity					
Mild stenosis (n,%)	269 (39.0)	322 (43.3)	252 (34.2)	211 (30.3)^a^	< 0.001
Severe stenosis (n,%)	159 (22.9)	259 (34.8)^a^	270 (36.6)^a^	367 (52.7)^a^	< 0.001

Abbreviations: CAD, coronary artery disease; TyG, triglyceride-glucose; NLR, neutrophil-to-lymphocyte ratio; TC, total cholesterol; TG, triglycerides; HDL-C, high-density lipoprotein cholesterol; LDL-C, low-density lipoprotein cholesterol; FPG, fasting plasma glucose

Overall P value was for the test of difference among the four group

a, significantly different from low-TyG index + low-NLR group (the Bonferroni correction was applied)

### Analysis of the risk factors for CAD

Multivariate logistic regression analysis was performed for variables with significantly associated with CAD (P < 0.05) in univariate logistic regression. After adjusting for confounding factors (gender, age, smoking, hypertension and diabetes), the TyG index (odds ratio [OR] 1.250, 95% confidence interval [CI] 1.077–1.452) and NLR (OR 1.299, 95% CI 1.222–1.381) as continuous variables were independent risk factors for CAD (all P < 0.05).

Table 3 shows the combined value of TyG index with NLR level for predicting CAD. Patients with isolated high-NLR level (OR 1.400, 95% CI 1.238–1.583), isolated high-TyG (OR 1.366, 95% CI 1.251–1.492), and high-TyG combined with high-NLR (OR 1.418, 95% CI 1.119–1.797) were independently associated with CAD after adjusting for conventional confounders.

**Table 3:** Multivariate-adjusted OR and 95% CI for CAD

Groups	OR (95%CI)^a^	P Value
low-TyG and low-NLR	1.00 (Reference)	
low-TyG and high-NLR	1.400 (1.238–1.583)	< 0.001
high-TyG and low-NLR	1.366 (1.251–1.492)	< 0.001
high-TyG and high-NLR	1.418 (1.119–1.797)	0.004

Abbreviations: CAD, coronary artery disease; TyG, triglyceride-glucose; NLR, neutrophil-to-lymphocyte ratio; OR: odds ratio; CI: confidence interval

a, adjustment for age, gender, smoking, hypertension, diabetes, TC, TG, HDL-C, LDL-C

### Diagnostic ability of TyG, NLR, and their combination in predicting CAD

**Fig. 1:**
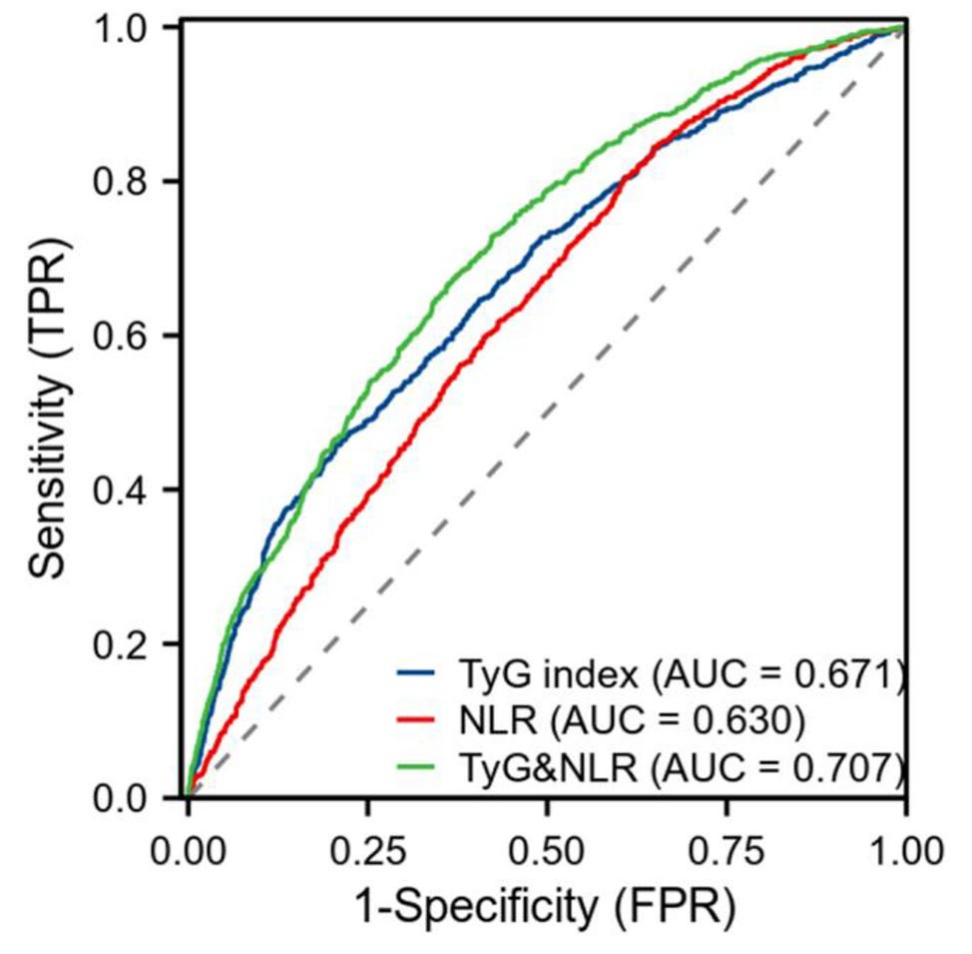
ROC diagram of TyG, NLR, and their combination in predicting CAD

Abbreviations: CAD, coronary artery disease; TyG, triglyceride-glucose; NLR, neutrophil-to-lymphocyte ratio; ROC: receiver operating characteristic; AUC: area under the receiver operating characteristic; FPR: false positive rate; TPR: true positive rate.

As shown in Fig. 1, the area under the receiver operating characteristic (ROC) curve (AUC) of TyG, NLR, TyG combined with NLR for predicting CAD were 0.671 (95% CI 0.648–0.694), 0.630 (95% CI 0.608–0.652), 0.707 (95% CI 0.685–0.728), respectively. Thus, the combination of the TyG index and NLR was superior to TyG index or NLR in predicting CAD.

### Baseline characteristics according to CAD severity

Table 4 shows baseline characteristics of patients in different groups according to the severity of CAD. Compared with the mild stenosis CAD group, the severe stenosis CAD group had higher TyG index and NLR values (all P < 0.05).

**Table 4:** Baseline characteristics of CAD patients according to CAD severity

Characteristics	Mild stenosis (n = 1054)	Severe stenosis (n = 1055)	X^2^/t/Z	P Value
Male (n,%)	707 (67.1)	760 (72.0)	6.126	0.013
Age (years)	62.26 ± 9.98	63.06 ± 10.10	-1.822	0.069
Smoking (n, %)	379 (36.0)	410 (38.9)	1.899	0.168
Hypertension (n, %)	625 (59.3)	738 (70.0)	26.183	< 0.001
Diabetes (n, %)	253 (24.0)	346 (32.8)	20.044	< 0.001
NE counts (×10^9^/L)	4.30 ± 1.98	5.14 ± 3.85	-6.309	< 0.001
LY counts (×10^9^/L)	1.61 ± 0.57	1.61 ± 0.62	-0.189	0.850
TC (mmol/L)	4.13 ± 0.95	4.60 ± 1.21	-9.808	< 0.001
TG (mmol/L)	1.61 ± 1.23	2.04 ± 1.72	-6.571	< 0.001
HDL-C (mmol/L)	1.09 ± 0.28	1.05 ± 0.25	4.074	< 0.001
LDL-C (mmol/L)	2.48 ± 0.80	2.89 ± 0.99	-10.566	< 0.001
FPG (mmol/L)	5.97 ± 2.02	6.46 ± 2.36	-5.200	< 0.001
TyG index	8.75 ± 0.58	8.99 ± 0.67	-8.896	< 0.001
NLR	3.06 ± 2.23	3.87 ± 2.81	-5.923	< 0.001
Gensini score	18.3 (13.0,22.0)	40.5 (30.5,70.0)	-39.763	< 0.001

Abbreviations: CAD, coronary artery disease; TyG, triglyceride-glucose; NLR, neutrophil-to-lymphocyte ratio; NE, neutrophil; LY, lymphocyte; TC, total cholesterol; TG, triglycerides; HDL-C, high-density lipoprotein cholesterol; LDL-C, low-density lipoprotein cholesterol; FPG, fasting plasma glucose

### Analysis of the risk factors for severe stenosis in CAD

The severity of CAD (mild or severe stenosis CAD) was used as dependent variable, multivariate logistic regression analysis was performed for variables with significantly associated with CAD severity (P < 0.05) in univariate logistic regression. The results showed that the TyG index (OR 1.811, 95% CI 1.561–2.102) and NLR (OR 1.129, 95% CI 1.088–1.172) as continuous variables were independent risk factors for severe stenosis in CAD (all P < 0.05).

Table 5 shows the combined value of TyG index with NLR level for predicting severe stenosis in CAD. Patients with isolated high-NLR level (OR 1.158, 95% CI 1.017–1.319), isolated high-TyG (OR 1.393, 95% CI 1.275–1.522), and high-TyG combined with high-NLR (OR 1.692, 95% CI 1.296–2.211) were independently associated with severe stenosis in CAD after adjusting for conventional confounders.

**Table 5:** Multivariate-adjusted OR and 95% CI for severe stenosis CAD

Groups	OR (95%CI)^a^	P Value
low-TyG and low-NLR	1.00 (Reference)	
low-TyG and high-NLR	1.158 (1.017–1.319)	0.026
high-TyG and low-NLR	1.393 (1.275–1.522)	< 0.001
high-TyG and high-NLR	1.692 (1.296–2.211)	< 0.001

Abbreviations: CAD, coronary artery disease; TyG, triglyceride-glucose; NLR, neutrophil-to-lymphocyte ratio; OR: odds ratio; CI: confidence interval

a, adjustment for gender, smoking, hypertension, diabetes, TC, TG, HDL-C, LDL-C

### Diagnostic ability of TyG, NLR, and their combination in predicting severe stenosis in CAD

**Fig. 2:**
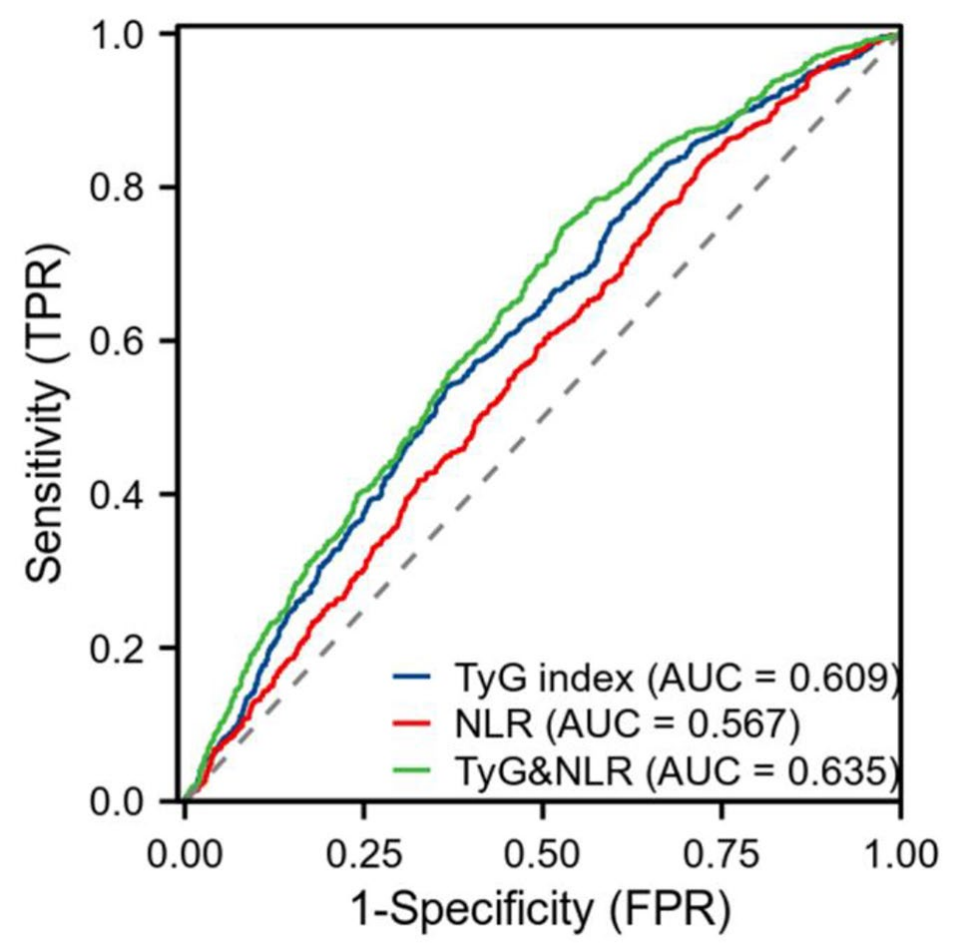
ROC diagram of TyG, NLR, and their combination in predicting severe stenosis CAD

Abbreviations: CAD, coronary artery disease; TyG, triglyceride-glucose; NLR, neutrophil-to-lymphocyte ratio; ROC: receiver operating characteristic; AUC: area under the receiver operating characteristic; FPR: false positive rate; TPR: true positive rate.

As shown in Fig. 2, the AUC of TyG, NLR, TyG combined with NLR for predicting severe stenosis in CAD were 0.609 (95% CI 0.585–0.633), 0.567 (95% CI 0.542–0.591), 0.635 (95% CI 0.611–0.658), respectively. Thus, the combination of the TyG index and NLR was also superior to TyG index or NLR in predicting severe stenosis in CAD.

## Discussion

CAD is the second leading cause of death in the Chinese population, and its prevalence and mortality are increasing year by year [2, 12]. Atherosclerosis is the main pathophysiological basis of CAD, and inflammation plays a pivotal role in the entire process of atherosclerosis [4, 13]. Neutrophils, the important inflammatory cells, have been regarded as important players in atherosclerosis-related inflammation [13]. A high level of circulating neutrophils can cause a hypercoagulable state by increasing blood viscosity, and lead to microvascular injury and reperfusion injury by interacting with platelets and vascular endothelial cells [14]. It has been proved that neutrophil counts were related to the extent of atherosclerosis and were positively correlated with the sizes of atherosclerotic lesions [15, 16]. In contrast, lymphocytes, the immune regulatory cells, have antiatherosclerotic effects. Inflammatory mediators from lymphocytes have a modulatory effect on neutrophils and inflammatory process. Oxidative stress plays an important role in the pathological process of atherosclerosis. The number of lymphocyte counts will decrease when oxidative stress occurs. NLR is a new measure of system inflammation, which integrates neutrophils and lymphocytes. Unlike other inflammatory biomarkers, such as hsCRP and IL-6 which are not detected routinely, NLR is convenient and easy to obtain in daily clinical work. NLR has been proposed as a useful diagnostic and prognostic marker for CAD [17]. A meta-analysis of 17 studies involving 7017 patients with CAD showed that NLR was independently associated with CAD severity and confirmed the diagnostic power of NLR in predicting severe stenosis in CAD [18].

In addition to inflammation, IR is another factor that plays an important role in the development and progression of atherosclerosis [19]. IR has a strong relationship with atherosclerotic cardiovascular diseases, especially CAD [20–22]. IR refers to the decreased efficiency of insulin-mediated glucose uptaking and utilizating, that is, the sensitivity and responsiveness of insulin cells to insulin are reduced. IR cause glucose and lipid metabolism disorders, inflammatory reactions, thrombosis, endothelial dysfunction, which ultimately leads to atherosclerosis [21]. The traditional “gold standard” for evaluating insulin resistance is the hyperinsulinemic-euglycemic clamp test. However, it is difficult to apply the hyperinsulinemic-euglycemic clamp test in large-scale studies because it is expensive, time-consuming and difficult to perform. TyG index, which combines triglyceride and fasting blood glucose, can be used as a reliable surrogate index to assess IR [23]. A previous study showed that the TyG index had high sensitivity (96.5%) and specificity (85.0%) for detecting IR compared with the hyperinsulinemic-euglycemic clamp test [24]. Multiple studies have demonstrated that TyG index is associated with cardiovascular disease [25, 26]. At present, there are also studies focusing on the relationship between TyG index and CAD. Park et al. found that TyG index was an independent predictor of CAD in people without traditional cardiovascular risk factors [27]. In addition, a prospective study with more than 10 years of follow-up found that TyG index was associated with an increased risk of CAD, whether as a continuous or categorical variable [28]. Recently, two large sample retrospective studies in China showed that TyG index was associated with the severity of CAD and could be used as a predictor of CAD severity [29, 30].

Inflammation and IR are important predisposing factors for the development of atherosclerosis. Inflammation and IR are also intimately related in atherosclerosis. Under normal conditions, the binding of insulin to the insulin receptor stimulates tyrosine autophosphorylation of the receptor, which then phosphorylates adaptor proteins such as insulin receptor substrates (IRS). IRS can activate phosphatidylinositol 3-kinase and pyruvate dehydrogenase kinase 1 to transduce signals to protein kinase B and glucose transporter (GLUT). GLUT-4 vesicles are transported to the plasma membrane, which turn on glucose transport. Inflammatory mediator can impaire insulin signaling by several mechanisms and result in IR [7]. Inflammatory factors can also aggravate IR by inducing oxidative stress. Furthermore, inflammation was shown to be driven by IR and IR can increase the intensity of inflammation [31]. In summary, the relationship between inflammation and IR is bidirectional with each promoting the other, and eventually jointly contribute to atherosclerosis.

NLR and TyG index, as indicators of inflammation and IR, have become important predictors of CAD and adverse events. However, there is a lack of research on the combined value of TyG index and NLR in predicting CAD and CAD severity. Therefore, our study firstly investigated the combined value of TyG index and NLR in predicting CAD and CAD severity.

In this study, multivariate logistic regression analysis showed that TyG index and NLR were independent risk factors for CAD and severe stenosis in CAD (P < 0.05), which was consistent with previous studies. Compared with the low-TyG and low-NLR group, patients with high-TyG and high-NLR group had a 1.418 times higher OR of having CAD and a 1.692 times higher OR of having severe stenosis in CAD in the multivariable logistic regression model. Notably, the OR values of the high-TyG and high-NLR group were higher than those of the isolated high-NLR group and the isolated high- TyG group. ROC curve analysis showed that the combination of the TyG index and NLR had a higher diagnostic efficiency for CAD and CAD severity than TyG index or NLR alone.

However, there are some limitations in our study. First, the most important limitations of the study is that it is a retrospective study. So, we can not prove a causal association of TyG index and NLR with CAD. Second, we only collected the data from a single hospital. Third, the participants were only inpatients in a Chinese hospital. Therefore, multi-center, large-sample, prospective studies are needed to further explore the association of TyG index and NLR with CAD.

## Conclusions

In conclusion, this study found that both high-TyG and high-NLR level were independently associated with CAD and CAD severity. In addition,this is the first study to indicate that the combination of the TyG index and NLR was superior to TyG index or NLR in predicting CAD and severe stenosis in CAD.

## Data Availability

The data used and analyzed to support the findings of this study are available from the corresponding author on reasonable request.

## Abbreviations

TyG: Triglyceride-glucose
NLR: Neutrophil-to-lymphocyte ratio
CAD: Coronary artery disease
CAG: Coronary angiography
IR: Insulin resistance
NE: Neutrophil
LY: Lymphocyte
FPG: Fasting plasma glucose
TC: Total cholesterol
TG: Triglycerides
HDL-C: High-density lipoprotein cholesterol
LDL-C: Low-density lipoprotein cholesterol
OR: Odds ratio
CI: Confdence interval
ROC: Receiver operating characteristic
AUC: Area under the receiver operating characteristic
FPR: False positive rate
TPR: True positive rate
IRS: Insulin receptor substrates
GLUT: Glucose transporter
